# Histomorphometric Evaluation of Gingival Phenotypic Characteristics: A Cross-Sectional Study

**DOI:** 10.3390/dj13080350

**Published:** 2025-07-31

**Authors:** Dimitrios Papapetros, Karin Nylander, Sotirios Kalfas

**Affiliations:** 1 Department of Preventive Dentistry, Periodontology and Implant Biology, Dental School, Aristotle University of Thessaloniki, 54124 Thessaloniki, Greece; kalfas@dent.auth.gr; 2 Department of Medical Biosciences, Umeå University, SE-90185 Umeå, Sweden; karin.nylander@umu.se

**Keywords:** gingiva, alveolar mucosa, phenotype, histology, thickness, keratinized tissue width, keratinization

## Abstract

**Objectives**: This study aims to explore the histological dimensions of the gingiva and the alveolar mucosa and to evaluate their associations with gingival phenotypic parameters, including gingival thickness (GT), keratinized tissue width (KTW), and gingival transparency. **Methods:** Histological and clinical assessments were performed on 45 healthy volunteers. Gingival and mucosal tissue samples were collected from the mucogingival junction region of one maxillary central incisor. Histomorphometric analysis included measurements of gingival and mucosal thickness, epithelial thickness, connective tissue thickness, epithelial papilla length and density, and keratinization. Clinical parameters included KTW and probe visibility upon insertion into the gingival sulcus. Correlations were statistically analyzed between clinical and histological parameters. **Results:** Probe visibility showed no significant correlations with any assessed parameter. Histological gingival thickness strongly correlated with gingival connective tissue thickness, moderately with epithelial thickness and papilla length, and weakly with papilla density. Mucosal thickness was strongly associated with connective tissue thickness and moderately with keratinization, but not with other parameters. KTW exhibited weak correlations with epithelial thickness and papilla length. **Conclusions:** Variability in gingival and mucosal thickness is primarily determined by connective tissue thickness, with a smaller contribution from the epithelium. Increased thickness is associated with longer, sparser epithelial papillae and with a tendency toward higher keratinization. KTW is significantly associated with epithelial thickness and papilla length, underscoring its relevance in gingival phenotype characterization.

## 1. Introduction

The gingiva plays a critical role in maintaining oral health by acting as a protective barrier against mechanical, microbial, and chemical challenges. Its anatomical and histological characteristics significantly influence its clinical presentation and function, particularly regarding shape, volume, and texture. These features collectively define gingival phenotypes, which are recognized as clinically relevant factors associated with periodontal stability, and treatment outcomes [1,2,3,4,5].

Various methods incorporating different parameters have been employed to define gingival phenotypes, leading to variability in both the number and categorization of phenotype groups. Simple clinical approaches include visual inspection and the evaluation of gingival transparency via insertion of a periodontal probe into the gingival sulcus [6,7,8]. More advanced methods, such as complex cluster analysis, have been used to classify phenotypes based on multiple clinical and radiographic parameters [6,9,10,11]. Among these, gingival thickness is widely regarded as the primary determinant of gingival phenotype. Accordingly, several studies have attempted to establish threshold values for gingival thickness corresponding to distinct phenotypic categories [12,13]. However, despite substantial research efforts, standardized criteria for gingival phenotype classification have yet to be established [11,13].

The 2017 World Workshop on the Classification of Periodontal and Peri-Implant Diseases and Conditions recognized three gingival phenotypes: thin scalloped, thick scalloped, and thick flat [14]. This classification was primarily based on gingival thickness and the width of keratinized tissue (KTW), highlighting their importance in clinical diagnosis and treatment planning. However, the underlying histological characteristics associated with these phenotypes remain only partially understood. Although soft tissue dimensions have been linked to the success of surgical and restorative procedures, the precise relationships between clinical parameters and histomorphometric features—such as connective tissue volume, epithelial thickness, and the degree of keratinization—have not been fully elucidated. Additionally, while the oral mucosa shares structural similarities with gingival tissue, it may display distinct patterns in the relationship between clinical appearance and histological composition.

The morphological and textural variability observed in the gingiva suggests corresponding differences in its underlying histological architecture; however, available data on this relationship remain limited. A previous study [15] identified two distinct types of gingival connective tissue—loose and dense—characterized by differing proportions of collagen types I and III. Another study [16] investigating the relationship between gingival phenotype and histological dimensions reported that the thick phenotype, as determined by transparency assessment, exhibited significantly greater gingival thickness, attributed solely to an increase in connective tissue thickness. Beyond these findings, the literature offers limited insight into the associations between histomorphometric parameters and clinically observed gingival morphology. This gap highlights the need for a more integrated approach to better understand the link between clinical assessments and gingival histology.

Gingival transparency is widely used as a simple, non-invasive clinical method for estimating gingival phenotype. This parameter is typically assessed at the sulcus and predominantly reflects the thickness of the gingival margin. However, there is currently no conclusive evidence that such a localized measurement accurately represents phenotypic characteristics across the full apico-coronal dimension of the gingiva. The correlation between gingival transparency and histological features in more apical regions of the soft tissue has not yet been thoroughly investigated. A comprehensive understanding of gingival characteristics in these apical areas is particularly critical in the context of surgical procedures involving flap elevation, where flap thickness has been associated with clinical outcomes in periodontal plastic surgery [17].

Accordingly, the present study was designed to investigate the histological dimensions of the gingiva and mucosa, and their associations with clinically relevant parameters, including gingival thickness, keratinized tissue width, and gingival transparency.

## 2. Materials and Methods

This cross-sectional study included 45 healthy adults (≥18 years old) from a Greek population and was conducted at the Department of Preventive Dentistry, Periodontology, and Implant Biology, School of Dentistry, Aristotle University of Thessaloniki, between 5 March 2020 and 1 December 2022. The required sample size was calculated based on a previous study [18] that employed the probe visibility technique and reported a correlation coefficient of 0.42 between gingival thickness and phenotypic classification (thin vs. thick). Assuming a significance level of 0.05 and a statistical power of 0.8, power analysis indicated that a minimum of 33 participants would be necessary.

The study was approved by the Ethics Committee of the Dental School of the Aristotle University of Thessaloniki, Greece (Approval number: 8/03.07.2019), and was performed in accordance with the Declaration of Helsinki. Prior to enrolment, all participants were informed about the objectives and the procedures of the study and provided written informed consent.

Inclusion criteria required the presence of intact anterior maxillary teeth and a keratinized tissue width of at least 3 mm to ensure that a minimum of 1 mm of intact gingiva would remain following biopsy collection. Participants were excluded if they exhibited probing depths exceeding 2 mm, clinical attachment loss, gingivitis, altered passive eruption, gingival pigmentation, trauma, or any pathological condition in the maxillary incisor region. Further exclusion criteria included tooth crowding, abnormal tooth angulation, dental abrasion, or restorations on the buccal surface of maxillary incisors, as well as a history of periodontal surgery or orthodontic treatment in the anterior maxilla. Additional exclusions encompassed systemic conditions or medications that could affect soft tissue metabolism, heavy smoking (defined as ≥10 cigarettes per day), and pregnancy or lactation.

### 2.1. Clinical Assessments

**Figure 1:**
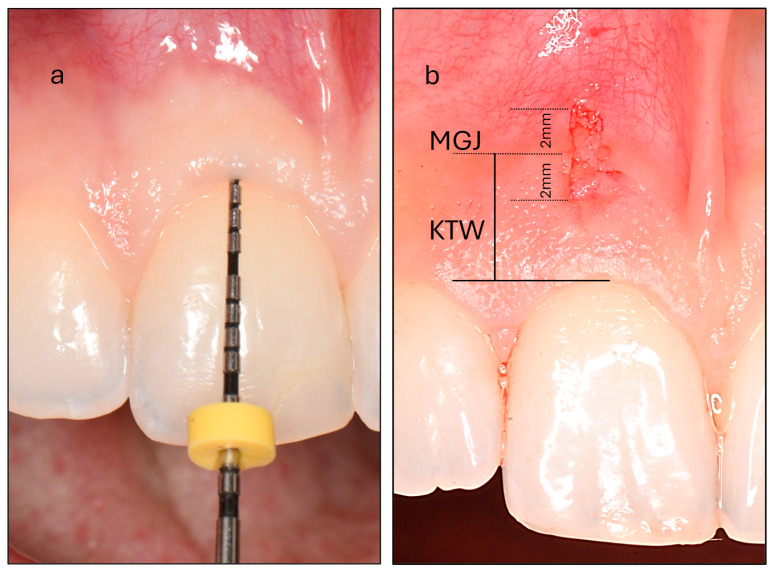
(**a**) Assessment of probe visibility was performed by inserting a periodontal probe into the gingival sulcus of the maxillary central incisor. In this example, the probe is completely concealed, classifying the participant as belonging to the thick phenotype group. (**b**) Measurement of the keratinized tissue width (KTW) at the maxillary central incisor. The mucogingival junction (MGJ) bisects the biopsy area. The biopsy specimen measures 4 mm in vertical length and 1 mm in horizontal width, extending 2 mm on either side of the MGJ.

The following clinical assessments were conducted in the region of the right or left maxillary central incisor during a single appointment by an experienced and previously calibrated examiner (D.P.):

(a) Probe Visibility (PV): Participants were categorized into thin and thick PV groups based on the visibility of a periodontal probe through the gingiva after its insertion into the gingival sulcus (Figure 1). Intra-examiner reproducibility was evaluated prior to the study through duplicate assessments of thirty maxillary incisors performed 15 days apart, yielding a high Cohen’s kappa coefficient of 0.870.
(b) Keratinized Tissue Width (KTW): This was measured as the distance between the mucogingival junction (MGJ) defined using the wrinkling method and the zenith of the free gingiva of one maxillary central incisor. A periodontal probe was positioned parallel to the sagittal plane, and a standardized photograph was taken with a camera aligned to the participant’s frontal plane. The image was subsequently analyzed using specialized image analysis software (ImageJ) and KTW was digitally measured following calibration (Figure 1).

### 2.2. Histological Assessments

Local anesthesia (2% lidocaine hydrochloride with 1:100,000 epinephrine; Lignospan standard, Septodont, Saint-Maur-des-Fossés, France) was administered prior to collecting a full-thickness soft tissue sample measuring 1 × 4 mm from the buccal region of the right maxillary incisor in each participant. Two parallel incisions, spaced 1 mm apart, were made and extended 2 mm on either side of the mucogingival junction. These incisions were joined by two perpendicular cuts to create a rectangular tissue sample (1 × 4 mm) (Figure 1). The sample was gently separated from the underlying bone using a papilla elevator (PH26M, Hu-Friedy, Chicago, IL, USA). Following biopsy collection, hemostasis was achieved by applying firm pressure with sterile gauze for approximately two minutes. Participants were instructed to rinse twice daily with 0.12% chlorhexidine gluconate for 7 days to support plaque control and prevent local infection. For pain management, 600 mg of ibuprofen was recommended as needed, not to exceed the maximum daily dosage. Patients were also advised to avoid mechanical trauma to the biopsy site during the initial healing phase. A clinical re-evaluation was performed 7 to 10 days postoperatively to monitor healing and ensure the absence of complications.

**Figure 2:**
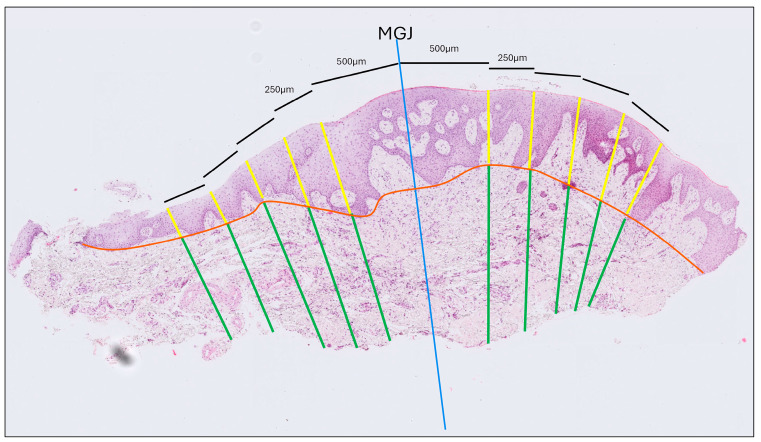
Histological thickness measurement of a tissue sample stained with hematoxylin and eosin. The blue line indicates the mucogingival junction (MGJ). Thickness was measured at five locations—0.5, 0.75, 1.0, 1.25, and 1.5 mm from the MGJ—on both the gingival and the mucosal sides. The orange line marks the interface between the epithelium and connective tissue. Yellow lines denote epithelial thickness measurements, while green lines correspond to connective tissue thickness measurements.

**Figure 3:**
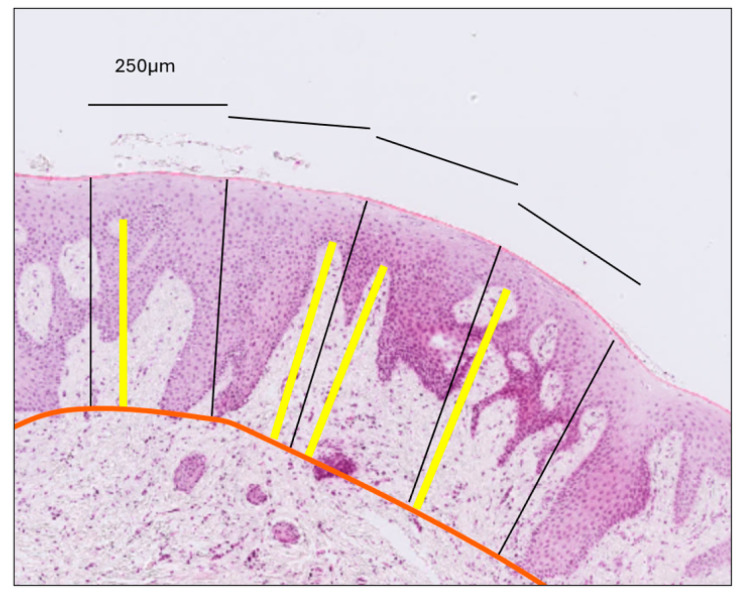
Measurement of connective tissue papilla length. The orange line delineates the interface between the epithelium and connective tissue. Perpendicular black lines divide the epithelium into compartments, each with a surface length of 250 μm. Within each compartment, the yellow line—drawn perpendicular to the gingival surface—extends from the tip of the longest papilla to the epithelial–connective tissue boundary. Papilla length was calculated as the average length of the yellow lines across all compartments, separately for the gingiva and the mucosa.

**Figure 4:**
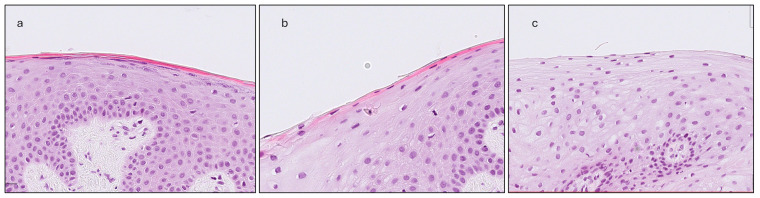
The three levels of keratinization assessment: (**a**) Ortho-keratinization; well-defined acellular keratin layer. (**b**) Para-keratinization; keratin layer includes cells that retain their nuclei. (**c**) Non-keratinization; no keratin layer present, superficial cells with their nuclei.

The harvested tissue samples were fixed in 10% buffered formalin, dehydrated, and embedded in paraffin. Serial 5-micrometer sections were prepared from the embedded tissue, stained with hematoxylin and eosin, and scanned using a digital slide scanner (NanoZoomer 2.0HT, Hamamatsu Photonics, Hamamatsu, Japan). On each scanned image, a perpendicular line was drawn around the midpoint of the sample, corresponding to the transition point in keratinization levels. This line divided the sample into two sections: the gingival and mucosal parts. Image analysis software (QuPath 3.0) was used to perform the following assessments:

(a) Gingival Thickness (GT): Defined as the length of a line drawn perpendicular to the epithelial surface, extending from the epithelial surface to the excised surface of the connective tissue and calculated as the mean of five measurements taken at 500, 750, 1000, 1250 and 1500 μm coronally to the MGJ (Figure 2).
(b) Gingival Epithelium Thickness (G-Ep): Calculated by averaging the lengths of the epithelial portions of the previously drawn lines. For this measurement, a line connecting the deepest points of the epithelial projections was drawn to delineate the boundary between the epithelium and connective tissue (Figure 2).
(c) Gingival Connective Tissue Thickness (G-CT): Measured using the same set of lines as the average length of the portions extending from the excised surface of the connective tissue to the epithelial boundary line (Figure 2).
(d) Gingival Papilla Length (G-P): Assessed as the average length of the longest papillae observed within each epithelial compartment, as defined by the previously established perpendicular lines (Figure 3).
(e) Gingival Papilla Density (G-Pd) (number/mm): Assessed as the number of clearly identifiable epithelial papillae divided by the total length of the epithelial surface.
(f) Gingival Keratinization (G-Ker): Assessed as the highest level of keratinization observed across the superficial layer of the gingiva epithelium. The levels recorded were defined as follows: ortho-keratinization, when a well-defined keratin layer with anucleate cells was observed; para-keratinization, when a keratin layer with flattened cells that retained their nuclei were observed; and non-keratinization, when a keratin layer was not observed, and superficial cells retained their shape and nuclei (Figure 4).

The same methodology was applied to the mucosal portion of each biopsy to assess mucosal thickness (MT), mucosal epithelium thickness (M-Ep), mucosal connective tissue thickness (M-CT), mucosal papilla length (M-P), mucosal papilla density (M-Pd), and mucosal keratinization (M-Ker). All dimensional measurements were recorded in millimeters.

### 2.3. Statistical Analysis

Descriptive statistics were presented as means and standard deviations. The Shapiro–Wilk test was applied to evaluate the normality of variable distributions. Pearson’s correlation coefficient and Spearman’s rank correlation were used to explore associations between normally and non-normally distributed variables, respectively. Pearson’s point-biserial correlation was employed to assess relationships involving binary and continuous variables. The chi-square test was used to analyze the association between PV groups and keratinization levels. For comparing the mean values of continuous variables between two groups, independent samples *t*-tests were applied for normally distributed data, while Mann–Whitney U tests were used for non-normally distributed data. Paired-sample *t*-tests were conducted to compare histological measurements between mucosal and gingival tissues. Statistical analyses were performed using SPSS software (Version 27, SPSS Inc., Chicago, IL, USA).

## 3. Results

### 3.1. Descriptive Statistics

A total of 45 individuals participated in the study. Histological measurements were performed on 36 samples, while 9 were excluded due to significant loss of tissue, distortion, or poor orientation. No adverse healing events were reported at the tissue sampling sites during the one-week or one-month follow-up assessments. Baseline demographic data for the entire cohort and each PV group are presented in Table 1.

Descriptive statistics for the clinical and histological variables, both for the total sample and stratified by PV group, are provided in Table 2. No statistically significant differences were detected between PV groups for any histological variable.

The differences in histological parameters between gingiva and mucosa are summarized in Table 3. The gingiva demonstrated significantly greater mean values in overall tissue thickness, epithelial thickness, papilla length, and papilla density (*p* ≤ 0.003). However, no significant difference was observed in connective tissue thickness (*p* = 0.301).

**Figure 5:**
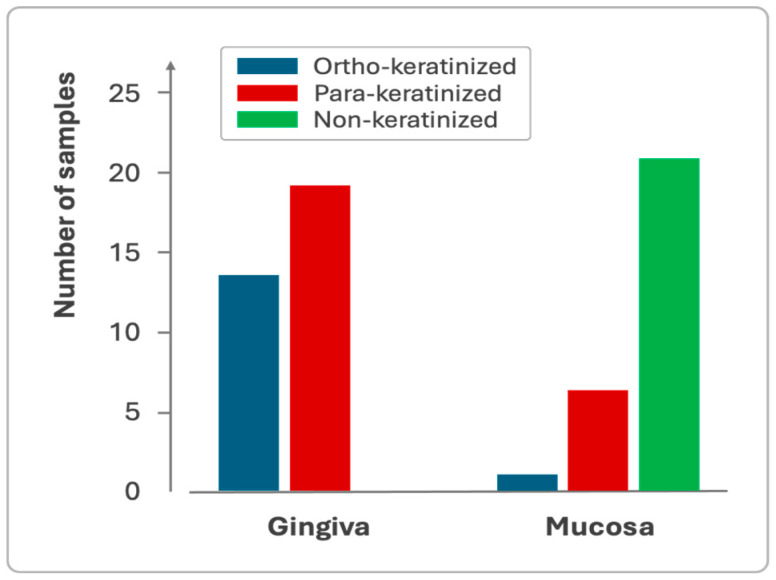
Sample distribution based on the keratinization profile of the gingival and mucosal epithelium.

Gingiva and mucosa showed distinct keratinization profiles, with the gingiva exhibiting both para-keratinized and ortho-keratinized samples, while the mucosa predominantly displayed non-keratinized epithelium (Figure 5).

### 3.2. Correlations

All correlations among the examined variables are presented in Table 4. No statistically significant associations were found between PV and any histological parameter (*p* ≥ 0121). GT exhibited a strong positive correlation with G-CT (r = 0.917, *p* < 0.001), as well as moderate and statistically significant correlations with all other gingival histological parameters (*p* ≤ 0.036). MT showed a strong positive correlation with M-CT (r = 0.944, *p* < 0.001), but did not exhibit significant associations with other mucosal dimensional parameters (r ≤ 0.109, *p* ≥ 0.581). MT also demonstrated significant correlations with GT (r = 0.602, *p* < 0.001) and G-CT (r = 0.716, *p* < 0.001). KTW displayed weak yet statistically significant positive correlations with G-Ep (r = 0.386, *p* = 0.020) and G-P (r = 0.351, *p* = 0.036), while no significant associations were observed with the remaining histomorphometric variables.

Regarding keratinization levels, GT showed a weak correlation with both G-Ker and M-Ker (r = 0.323 and r = 0.377, respectively), which was borderline significant for M-Ker (*p* = 0.046) and borderline non-significant for G-Ker (*p* = 0.062). MT showed a moderate, statistically significant positive correlation with M-Ker (r = 0.563, *p* = 0.002). KTW did not show any significant correlation with keratinization levels (*p* ≥ 0.607).

## 4. Discussion

The histological design of the present study provided valuable insights into the histomorphometry and structural organization of the gingiva and alveolar mucosa. In most specimens, tissue thickness progressively decreased from the gingival margin toward the mucosal edge, with a more pronounced reduction observed in the gingival region. This gradient was primarily attributed to variations in epithelial thickness, while connective tissue thickness remained relatively stable. Microscopically, tissue density appeared largely uniform within individual specimens, although the mucosal region generally exhibited a lower overall density. In some cases, increased connective tissue density was noted, characterized by broad, densely packed collagen bundles predominantly located in the superficial layer. These denser samples were frequently associated with above-average total tissue thickness, with collagen fibers extending beyond the mucogingival junction (MGJ) into the adjacent mucosa. Previous findings [15] have described two distinct connective tissue patterns in the gingiva, differing in collagen density and composition. These observations support the hypothesis that variations in overall tissue thickness may reflect shifts in the relative proportions and organization of histological components, potentially influencing the biomechanical and functional properties of the soft tissue.

A strong positive correlation was found between gingival thickness and connective tissue thickness, along with moderate correlations involving epithelial thickness and papilla length. These relationships highlight the interdependent structural dynamics underlying gingival phenotypes. The findings suggest that connective tissue thickness is the primary determinant of total gingival thickness, with epithelial thickness playing a secondary role. Increased gingival thickness was also associated with longer, less densely packed papillae, consistent with previous reports linking thick phenotypes to greater papillary dimensions [16]. The more pronounced interdigitation between epithelial rete pegs and connective tissue papillae observed in thicker gingiva may enhance biomechanical stability, potentially improving resistance to external forces and influencing clinical outcomes [19].

In the present study, no significant correlation was observed between probe visibility and any of the assessed histological parameters. Although the mean histologically measured gingival thickness was slightly greater in the probe-defined thick phenotype compared to the thin phenotype (1.09 mm vs. 1.00 mm, respectively), this difference was minimal and did not reach statistical significance. This contrasts with previous findings [16], which reported a 0.5 mm difference in gingival thickness between probe visibility-defined phenotypes, as determined by histological analysis. Furthermore, the absolute gingival thickness values in that study were considerably higher than those observed in the present investigation (1.9 mm for the thick phenotype and 1.4 mm for the thin phenotype). A likely explanation is the difference in biopsy location. In the aforementioned study, biopsies were reportedly obtained from the buccal aspect of anterior teeth; however, the specific teeth and the exact location on the buccal surface were not clearly defined. Based on published figures, sampling appears to have been performed near the gingival margin, in contrast to the more apical region examined in the present study. Given the known variability in gingival thickness along the buccolingual axis, such discrepancies in sampling location may significantly limit comparability [12,13].

Moreover, the gingival thickness values reported in that study [16] appear substantially elevated relative to those documented in other investigations. For instance, a CBCT-based investigation assessing gingival thickness at various apical levels in the maxillary anterior region reported mean values ranging from 0.95 to 1.23 mm [12], which are more consistent with the measurements observed in the present study. These findings further support the validity of our results. Additional methodological differences—such as variations in tissue sampling protocols, histological processing, measurement techniques, phenotype classification criteria and differences in study populations—may also account for the observed discrepancies. It is also important to note that the previous study [16] included only 10 participants (5 per phenotype group), which limits the accuracy and generalizability of its findings.

In the present study, biopsies were collected from sites located at least 1 mm apical to the gingival margin. This decision was guided by both ethical considerations—specifically, the need to avoid compromising gingival health and esthetics—and methodological constraints. Selecting marginal biopsy sites in individuals with varying keratinized tissue width might have led to inconsistent sampling levels across specimens, ranging from marginal to mid-gingival or even mucosal levels. To ensure anatomical consistency and enhance comparability between samples, we therefore standardized the biopsy location near the mucogingival junction.

Although this approach prevented direct sampling from the exact site where probe visibility is clinically assessed, it aligns with the broader anatomical concept of gingival phenotype, which encompasses the entire width of keratinized tissue. Probe visibility, while widely used as a simple clinical method to estimate phenotype, reflects only the marginal gingival thickness and may not accurately capture deeper structural features. The absence of significant correlations between probe visibility and histological parameters in our study suggests that this method may not reliably represent the underlying tissue architecture throughout the gingiva.

Keratinized tissue width (KTW) did not show a significant correlation with histological gingival or connective tissue thickness at the specific site assessed in this study. This contrasts with most previous reports, which have demonstrated positive correlations between KTW and gingival thickness at more coronal sites [12,20,21,22]. However, KTW was significantly associated with epithelial parameters, particularly epithelial thickness and papilla height, suggesting that it remains a clinically relevant indicator of gingival phenotype. To our knowledge, this is the first study to explore the relationship between KTW and the histomorphometric dimensions of the gingiva.

The alveolar mucosa was found to be thinner than the gingiva, primarily due to a reduction in epithelial thickness, while connective tissue thickness remained comparable between the two regions. This similarity likely reflects the close anatomical proximity of the sampled sites and may not be generalizable to comparisons with more distant anatomical regions. Previous CBCT-based studies have shown a gradual thinning of buccal soft tissues from the cementoenamel junction to 2 and 4 mm apically [10,12,23,24], followed by either a slight increase or stabilization at 6 mm [10,23,24]. However, direct comparison with our findings is limited, as those studies did not clearly define the location of the mucogingival junction. To the best of our knowledge, the present study is the first to investigate histological architecture at different vertical levels. In this context, mucosal thickness was strongly correlated with mucosal connective tissue thickness, while no significant associations were found with other dimensional parameters. These findings suggest that, similar to the gingiva, connective tissue plays a dominant role in determining overall mucosal thickness.

A weak, non-significant correlation was observed between gingival thickness and the degree of keratinization, whereas a moderate, statistically significant correlation was identified in the oral mucosa. No comparable data have been previously reported in the literature. Variations in keratinization may influence the texture and biomechanical properties of soft tissues; however, no differences in keratinization were detected between phenotype groups defined by probe visibility. The present study also revealed distinct keratinization patterns between the gingiva and mucosa: the gingiva predominantly exhibited para- and ortho-keratinization, whereas the mucosa was primarily non-keratinized, consistent with the functional specialization of these tissues [25]. Notably, the presence of keratinization in the mucosal region may reflect methodological influences, as the clinically defined mucogingival junction does not always coincide with its histological counterpart.

While this study provides novel insights into the histological characteristics of gingival and mucosal tissues, its limited sample size may not fully capture the variability of gingival phenotypes. It may also constrain the ability to detect strong associations between clinical and histological parameters. Larger cohort studies are needed to investigate gingival tissue architecture more comprehensively—not only in the region surrounding the mucogingival junction, but across the full width of the gingiva. Future studies should also incorporate multiple sampling sites across both the maxillary and mandibular arches to account for potential regional variations in gingival structure and phenotype distribution.

Moreover, technical limitations such as tissue shrinkage during histological processing, variability in sectioning planes, and potential exclusion of residual connective tissue adhering to the bone surface may contribute to underestimation of actual connective tissue thickness and compromise measurement accuracy. Future investigations should explore alternative or complementary methodologies to enhance the precision and reproducibility of histological assessments. Although the inclusion of sulcular and marginal gingiva would offer more comprehensive data on the structural correlates of clinically assessed phenotypes, such sampling was not feasible in the current study due to the risk of inducing gingival recession and compromising esthetics or periodontal health. For inherently subjective parameters such as probe visibility, the use of multiple independent evaluators is recommended to improve data reliability and minimize observer bias.

## 5. Conclusions

The findings of the present study highlight the central role of connective tissue in determining both gingival and mucosal thickness. Increased gingival thickness was associated with longer, broader connective tissue papillae, whereas greater keratinized tissue width (KTW) correlated with increased epithelial thickness and elongated papillae. Furthermore, greater overall tissue thickness was linked to higher levels of epithelial keratinization. These histological variations likely contribute to differences in the biomechanical and functional properties of gingival phenotypes, potentially influencing their clinical appearance and responsiveness to therapeutic interventions.

## Figures and Tables

**Table 1:** Demographic characteristics of the study participants, presented for the total sample and stratified by probe visibility (PV) groups.

	Total	Thin	Thick
Subjects	45	21	24
Age (mean, SD)	36.11 ± 10.9	37.33 ± 12.16	35.04 ± 9.81
Female/male	26/19	15/6	11/13
Smokers/non-smokers	13/32	4/17	9/15
Histological samples	36	17	19

**Table 2:** Descriptive statistics for clinical and histological variables, presented by PV group (thin and thick), along with the significance (*p*-value) of intergroup differences (Δ Thin–Thick).

	Total	Thin	Thick	Δ Thin–Thick
GT	1.05 ± 0.25	1.00 ± 0.22	1.09 ± 0.26	0.298
G-CT	0.68 ± 0.20	0.64 ± 0.15	0.72 ± 0.23	0.212
G-Ep	0.36 ± 0.09	0.35 ± 0.09	0.37 ± 0.10	0.626
G-P	0.26 ± 0.09	0.23 ± 0.08	0.27 ± 0.08	0.142
G-Pd	7.12 ± 1.59	7.38 ± 1.89	6.89 ± 1.28	0.368
MT	1.00 ± 0.23	0.94 ± 0.20	1.07 ± 0.25	0.128
M-CT	0.75 ± 0.23	0.69 ± 0.29	0.82 ± 0.27	0.121
M-Ep	0.25 ± 0.08	0.25 ± 0.08	0.25 ± 0.07	0.930
M-P	0.14 ± 0.07	0.14 ± 0.07	0.24 ± 0.07	0.635
M-Pd	5.70 ± 1.75	5.61 ± 1.66	5.79 ± 1.88	0.788
KTW	4.21 ± 1.00	3.90 ± 0.86	4.48 ± 1.06	0.023 *

GT, gingival thickness; G-CT, gingival connective tissue thickness; G-Ep, gingival epithelial thickness; G-P, gingival papilla length; G-Pd, gingival papilla density; MT, mucosal thickness; M-CT, mucosal connective tissue thickness; M-Ep, mucosal epithelial thickness; M-P, mucosal papilla length; M-Pd, mucosal papilla density; KTW, keratinized tissue width; *, statistically significant.

**Table 3:** Mean differences (Δ) and corresponding *p*-values for histological measurements comparing gingival and mucosal tissues.

Δ	Mean (mm)	*p*
GT–MT	0.09 ± 0.15	0.003 *
G-CT–M-CT	−0.03 ± 0.35	0.301
G-Ep–M-Ep	0.11 ± 0.08	<0.001 *
G-P–M-P	0.12 ± 0.08	<0.001 *
G-Pd–M-Pd (mm^−1^)	1.24 ± 1.76	<0.001 *

GT, gingival thickness; G-CT, gingival connective tissue thickness; G-Ep, gingival epithelial thickness; G-P, gingival papilla length; G-Pd, gingival papilla density; MT, mucosal thickness; M-CT, mucosal connective tissue thickness; M-Ep, mucosal epithelial thickness; M-P, mucosal papilla length; M-Pd, mucosal papilla density, *, statistically significant.

**Table 4:** Correlations between phenotypic parameters (PV, GT, MT, KTW) and histological variables.

	PV		GT		MT		KTW	
	r	*p*	r	*p*	r	*p*	r	*p*
GT	0.178	0.298	-	-	0.602	<0.001 *	0.276	0.187
G-CT	0.213	0.212	0.917	<0.001 *	0.716	<0.001 *	0.067	0.699
G-Ep	0.084	0.626	0.630	<0.001 *	0.350	0.067	0.386	0.020 *
G-P	0.249	0.142	0.737	<0.001 *	0.410	0.030 *	0.351	0.036 *
G-Pd	−0.154	0.368	−0.348	0.036 *	−0.029	0.996	−0.233	0.171
MT	0.295	0.128	0.602	<0.001 *	-	-	0.131	0.508
M-CT	0.300	0.121	0.725	<0.001 *	0.944	<0.001 *	0.149	0.450
M-Ep	0.001	0.997	0.234	0.222	0.109	0.581	0.066	0.733
M-P	−0.035	0.851	0.202	0.284	0.057	0.771	0.167	0.369
M-Pd	0.050	0.788	0.005	0.980	0.058	0.769	0.242	0.190
G-Ker	0.003	0.955	0.323	0.062	0.240	0.219	0.036	0.836
M-Ker	0.253	0.232	0.367	0.046 *	0.532	0.004 *	0.096	0.607

r, correlation coefficient; *p*, statistical significance; PV, probe visibility; GT, gingival thickness; G-CT, gingival connective tissue thickness; G-Ep, gingival epithelial thickness; G-P, gingival papilla length; G-Pd, gingival papilla density; MT, mucosal thickness; M-CT, mucosal connective tissue thickness; M-Ep, mucosal epithelial thickness; M-P, mucosal papilla length; M-Pd, mucosal papilla density; KTW, keratinized tissue width; G-Ker, gingival keratinization level; M-Ker, mucosal keratinization level; *, statistically significant.

## Data Availability

The data presented in this study are available from the corresponding author upon reasonable request.
